# Ascending Axonal Degeneration of the Corticospinal Tract in Pure Hereditary Spastic Paraplegia: A Cross-Sectional DTI Study

**DOI:** 10.3390/brainsci9100268

**Published:** 2019-10-09

**Authors:** Julia List, Zacharias Kohl, Juergen Winkler, Franz Marxreiter, Arnd Doerfler, Manuel A. Schmidt

**Affiliations:** 1 Departments of Molecular Neurology, University Hospital Erlangen, Friedrich-Alexander-University Erlangen-Nuremberg, Schwabachanlage 6, 91054 Erlangen, Germany; 2 Neuroradiology, University Hospital Erlangen, Friedrich-Alexander-University Erlangen-Nuremberg, Schwabachanlage 6, 91054 Erlangen, Germany

**Keywords:** hereditary spastic paraplegia, diffusion tensor imaging, fractional anisotropy, tract-based spatial statistics (TBSS), corticospinal tract

## Abstract

Objective: To identify structural white matter alterations in patients with pure hereditary spastic paraplegia (HSP) using high angular resolution diffusion tensor imaging (DTI). Methods: We examined 37 individuals with high resolution DTI, 20 patients with pure forms of hereditary spastic paraplegia and 17 age and gender matched healthy controls. DTI was performed using a 3 T clinical scanner with whole brain tract-based spatial statistical (TBSS) analysis of the obtained fractional anisotropy (FA) data as well as a region-of-interest (ROI)-based analysis of affected tracts including the cervical spinal cord. We further conducted correlation analyses between DTI data and clinical characteristics. Results: TBSS analysis in HSP patients showed significantly decreased fractional anisotropy of the corpus callosum and the corticospinal tract compared to healthy controls. ROI-based analysis confirmed significantly lower FA in HSP compared to controls in the internal capsule (0.77 vs. 0.80, *p* = 0.048), the corpus callosum (0.84 vs. 0.87, *p* = 0.048) and the cervical spinal cord (0.72 vs. 0.79, *p* = 0.003). FA values of the cervical spinal cord significantly correlated with disease duration. Conclusion: DTI metrics of the corticospinal tract from the internal capsule to the cervical spine suggest microstructural damage and axonal degeneration of motor neurons. The CST at the level of the cervical spinal cord is thereby more severely affected than the intracranial part of the CST, suggesting an ascending axonal degeneration of the CST. Since there is a significant correlation with disease duration, FA may serve as a future progression marker for assessment of the disease course in HSP.

## 1. Introduction

Hereditary spastic paraplegias (HSP) are comprised of a group of inherited neurodegenerative diseases characterized by a progressive weakness and spasticity of the lower limbs presumably caused by a dysfunction of motor neurons [1,2]. “Pure” forms of HSP presenting with isolated spastic paraparesis are distinguished from “complicated” forms with additional symptoms such as cerebellar ataxia, epilepsy, peripheral neuropathy, visual and cognitive impairment [3]. HSPs are rare diseases with an average prevalence of about 2–10/100,000 [4,5,6]. Until today, more than 80 genes and loci were identified and a wide phenotypic variability has been described [7,8,9].

Previous MRI studies in HSP revealed structural brain alterations like atrophy of the corpus callosum and cortical or cerebellar atrophy as well as increased white matter signal intensities particularly in patients with autosomal recessive complicated HSPs [10,11,12,13]. Using advanced imaging techniques (diffusion tensor imaging, DTI), broad changes of white matter in HSP patients have been revealed [14]. DTI analysis of HSP patients (SPG11, spastic paraplegia 11, classified as complicated HSP) has shown even more widespread alterations and atrophy of grey and white matter compared to conventional MR imaging in a small sample size [15,16]. One of the largest studies with 44 HSP patients revealed alterations of association and motor as well as cerebellar white matter tracts irrespective of genotype [14]. The changes in several non-motor regions led to the suggestion of a neurodegenerative pathway involving extramotor regions as well [14]. Additionally, a study with a small sample size found more severe changes in complicated HSP (SPG11) compared to pure HSP (SPG4) [17]. Important imaging findings in patients with SPAST (SPG4) mutations described white matter changes mainly in the corticospinal tract and the corpus callosum [18,19], in part additional grey matter atrophy [20]. Lindig et al demonstrated widespread affected grey and white matter including the corticospinal tract (CST), the corpus callosum, medio-dorsal thalamus, parieto-occipital regions, upper brainstem and the cerebellum in a cohort of 15 SPG4 patients [20].

Our aim was to identify microstructural alterations in relevant brain regions in patients with pure HSP (pHSP) using diffusion tensor imaging. We excluded complicated forms of the disease to avoid widespread changes of grey and white matter and performed reader independent, unbiased tract-based spatial statistics (TBSS) analysis followed by a region-of-interest (ROI)-based measurement of relevant brain regions.

More importantly, only a few previous studies analyzed the spinal cord of HSP patients, so far focusing on the volume-loss of the spinal cord without gaining information about the integrity of the CST [12,18,21]. As there is no specific tract-based analysis of the CST at the level of the cervical spinal cord of pHSP patients available, we further performed a ROI-based analysis of the lateral funiculus. As this method has not yet been performed in previous studies we aimed to quantify suspected impairment specifically of the cervical CST and to obtain new information consistent with possible pathophysiological theories.

## 2. Materials and Methods

### 2.1. Subjects

We recruited a total of 20 patients with pure hereditary spastic paraplegia (mean age 52.7 ± 9.7 years) and 17 age and gender matched healthy controls (mean age 55.0 ± 8.9 years). The clinical diagnosis was confirmed by an experienced movement disorder specialist according to the criteria of A. Harding [3] and genetic testing revealed mutations in SPG4, SPG5 and SPG31 in 13 subjects. A genetical confirmation of the disease in 7 patients was not possible. Patients underwent neurological examination and a full medical history. We used the spastic paraplegia rating scale (SPRS) to assess disease severity. Detailed clinical characteristics are shown in Table 1. The control subjects met the following inclusion criteria: No neurologic disease, no gait impairment, normal brain imaging without any structural damage.

This study was approved by the Clinical Investigation Ethics Committee of the University of Erlangen–Nuremberg. All subjects were informed in detail about the procedure and written informed consent was obtained from all participants.

### 2.2. MR Imaging

We performed high resolution MR scans of the brain and the cervical spinal cord of all subjects using a 3 Tesla scanner (Magnetom Tim Trio, Siemens Healthcare GmbH, Erlangen, Germany) with a gradient field strength up to 45 mT/m (72 mT/m effective). DTI was performed in the axial plane with 2 mm slice thickness using a single-shot, spin echo, echo planar imaging (EPI) diffusion tensor sequence (TR = 3400 ms, TE = 93 ms, FoV = 230 × 230 mm^2^, acquisition matrix size = 256 × 256 reconstructed to 512 × 512). Diffusion weighting was carried out with a maximal b-factor of 1000 s/mm^2^ along 15 icosahedral directions complemented by one scan with b = 0.

### 2.3. Tract-Based Spatial Statistics

**Figure 1:**
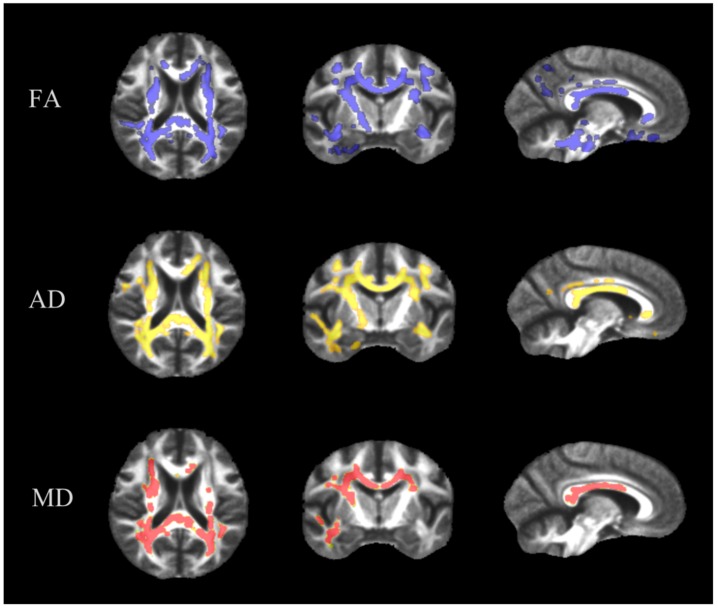
Whole brain voxel-wise analysis of white matter changes in HSP using tract-based spatial statistics (TBSS). TBSS of the brain of patients with pure HSP revealed reduced fractional anisotropy (FA, blue), increased axial diffusivity (AD, yellow) and increased mean diffusivity (MD, red) in comparison to controls. Displayed on mean FA template. Significant values (*p* < 0.05, cluster threshold correction for multiple comparison) are shown in blue, yellow and red respectively.

DICOM images were converted to NIfTI files (Neuroimaging Informatics Technology Initiative) using dcm2nii from the MRIcron package (http://www.mccauslandcenter.sc.edu/micro/mricron/dcm2nii.html). The resulting images were then preprocessed for TBSS analysis, i.e., corrected for eddy currents with eddy_correct and brain extracted with bet2 [22]. We chose a bet2 threshold of 0.2 to remove all non-brain tissue. Next, a diffusion tensor model was fitted at each voxel to extract the fractional anisotropy (FA) maps using DTIFIT. All of these preprocessing steps were carried out using the FMRIB Diffusion Toolbox (FDT), which is part of FSL (http://fsl.fmrib.ox.ac.uk/fsl) [23]. We used the JHU White-Matter Tractography Atlas for annotation [24,25]. For TBSS analysis, images were reviewed carefully and faulty images with quality issues were sorted out. Images were nonlinearly registered to the FMRIB58_FA standard template. An average mean FA map was created and the original FA images were nonlinearly registered to this group mean. This approach has been used before in TBSS analysis of patients with Alzheimer’s disease who had ventricular enlargement due to atrophy [26]. Axial diffusivity (AD) and mean diffusivity (MD) maps were calculated as well (Figure 1).

### 2.4. Region of Interest (ROI)-Based Analysis

To assess the extent of altered brain regions found in the TBSS analysis we performed region of interest based measurements. The analysis was carried out on a PACS workstation using syngo.via (Siemens Healthcare GmbH, Erlangen, Germany). For each measurement, the obtained FA maps were co-registered to an anatomical MPRAGE dataset. This procedure facilitated anatomical orientation in the colored FA maps and guaranteed correct placement of ROIs. Measurements of diffusion indices were carried out in the axial plane of the FA map on the slice the structure could be appreciated best. Circular ROIs were placed with a defined size depending on the target structure (corpus callosum splenium 20 mm^2^; rostrum 30 mm^2^; internal capsule 15–20 mm^2^; crus cerebri 10 mm^2^). The corpus callosum was analyzed at the level of the lateral ventricles. Three ROIs (left, middle, right) were drawn in the splenium and 3 ROIs in the rostrum of the corpus callosum. In the internal capsule, 3 ROIs were placed per side (anterior and posterior limb, genu). The crus cerebri was measured at the level of the mammillary bodies and the interpeduncular fossa (3 ROIs per side).

**Figure 2:**
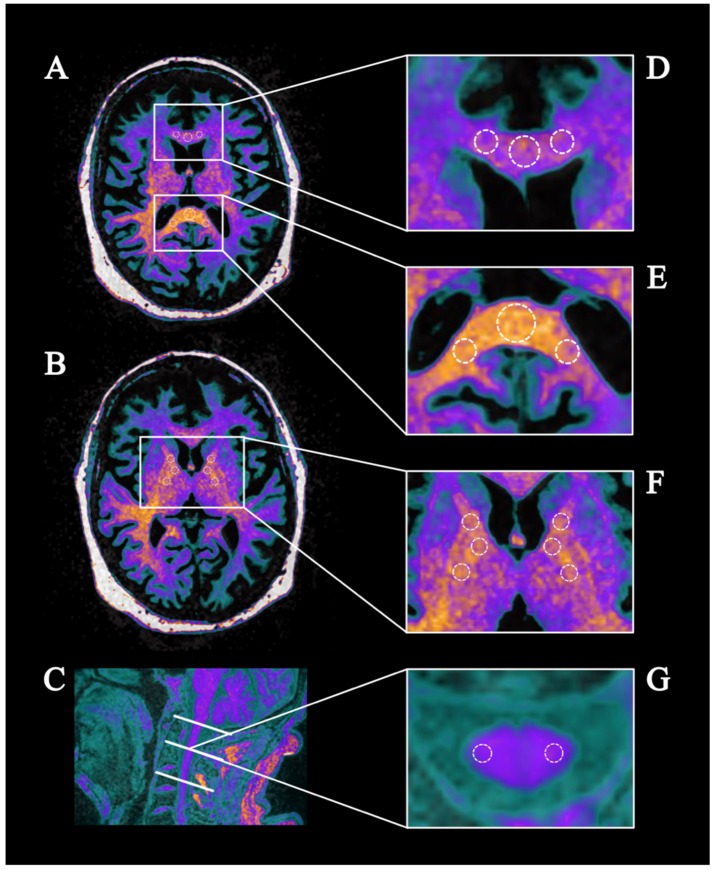
Regions of interest (ROIs) based analysis of the corpus callosum (A), capsula interna (B) and the cervical spinal cord (C) of HSP patients. In detail: ROIs in the rostrum (D) and splenium (E) of the corpus callosum, crus anterior, posterior and genu of the capsula interna (F) and the corticospinal tract of the cervical cord (G) at the level of the dens and the corpus of the second and third vertebral body (C).

Further measurements were performed at the upper cervical spinal cord at level of the dens and the center of the body of the second (C2) and third (C3) cervical vertebra. Similar to the analysis of the brain regions, colored FA maps were used. Sagittal co-registered MPRAGE datasets were used for identification of the correct area. Six ROIs of a total size of 7.5 mm^2^ each were drawn in the lateral funiculus bilaterally at each level. FA of each ROI was sumed up in mean values of each region (corpus callosum splenium and rostrum, internal capsule, cerebral crura, cervical spinal cord dens, second vertebra and third vertebra) for statistical analysis. ROI placement is depicted in Figure 2.

### 2.5. Statistical Analysis

Statistical analysis was performed using IBM SPSS Statistics for Macintosh (Version 25.0.0.1, IBM Corp. in Armonk, NY, United States). Kolmogorov-Smirnov test was performed to test for normal distribution of FA data. Depending on the distribution, data is presented as mean and standard deviation (SD).

Depending on the distribution two-samples t-tests or Mann-Whitney U tests were performed for group comparison of clinical data between the patient and the control group and in subgroup comparison between SPG4 and non-SPG4. Due to non-parametric data mean FA values between the patient and control group were analyzed using Mann-Whitney U tests. Age-matched controls were used due to an age-dependent change of white matter including the internal capsule and the corpus callosum [27]. Correlation analysis was carried out between DTI metrics and clinical data (age, disease duration and SPRS) using Pearson or Spearman correlation coefficients. TBSS data was corrected for multiple comparisons over space by controlling the family-wise error rate using threshold-free cluster enhancement. The significance level was set at *p* < 0.05 for all statistical analysis.

## 3. Results

### 3.1. TBSS

The unbiased TBSS analysis of the brain of pHSP patients revealed broad white matter alterations in multiple DTI indices including commissure, association and projection fibers compared to healthy controls (corrected for multiple comparisons, *p* < 0.05) as shown in Figure 1. Clusters of voxels with significantly reduced FA and increased AD and MD were detected in the corpus callosum and the centrum semiovale. Radial Diffusivity (RD) between pHSP patients and healthy controls did not differ significantly.

In detail, FA was reduced in white matter tracts including the corpus callosum, the corticospinal tract (centrum semiovale, the internal capsule, the crura cerebri), the optic radiation, the frontal lobe, parieto-occipital regions, the temporal lobe and the brainstem. FA of the cerebellum was not significantly different between patients and controls. AD was increased in areas with FA reduction. Highest values were detected in the subregions of the corpus callosum, the centrum semiovale, the inferior fronto-occipital fasciculus as well as the internal capsule on the right side. Similar to the FA results, AD of the cerebellum was not significantly different between patients and controls. MD was significantly increased in the body and splenium of the corpus callosum, the centrum semiovale, the right internal capsule and occipital regions. A subgroup comparison between the FA of SPG4 patients and non-SPG4 patients did not reveal any significant differences; therefore, no further analysis was performed.

### 3.2. ROI-Based Analysis

The subsequent ROI-based approach confirmed the results of the voxel-per-voxel analysis. FA data in the ROI-based analysis did not show a normal distribution; therefore, non-parametric comparison of mean values was performed. FA was reduced in descriptive statistical analysis in the internal capsule and the cerebral crura of the pHSP patients in comparison to the control group, but did not reach the significance level of *p* < 0.05. In contrast, the internal capsule, the corpus callosum and the cervical spinal cord showed significantly reduced FA compared to the controls. No further subgroup comparison regarding SPG4 subtype was performed as there was no significant difference found in the TBSS analysis between the SPG4 and the non-SPG4 patients. Age-matched controls were used due to an age-dependent change of white matter [27]. The results of the ROI-based analysis of the corpus callosum, the internal capsule, the cerebral crura and the cervical spinal cord are presented in Table 2.

### 3.3. ROI-Based Analysis of the Internal Capsule

FA was significantly reduced in the internal capsule (patients vs. controls 0.77 ± 0.06 vs. 0.80 ± 0.06, *p* = 0.048).

### 3.4. ROI-Based Analysis of the Corpus Callosum

**Figure 3:**
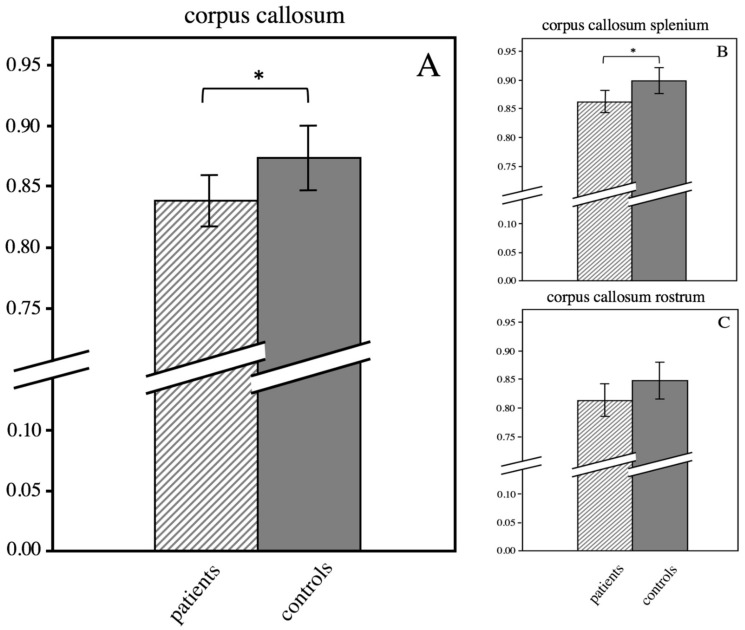
Decreased FA in the corpus callosum of HSP patients (cross-hatched bars) compared to controls (grey bars). HSP patients show significantly decreased FA values of the corpus callosum (A). Significantly decreased FA in the splenium of the corpus callosum (B), while FA of the rostrum (C) shows no significant difference. Error bars indicate 95% CI. Asterisk indicate *p* < 0.05.

Analyzing the FA values of the corpus callosum of pHSP patients revealed significant results (patients vs. controls 0.84 ± 0.05 vs. 0.87 ± 0.05, *p* = 0.048) in comparison to the control group (Figure 3A). While the differences of the FA values of the rostrum of the corpus callosum did not reach the significance level of *p* < 0.05 (Figure 3C), the splenium of the corpus callosum showed a significantly decreased FA value compared to controls (patients vs. controls 0.86 ± 0.04 vs. 0.90 ± 0.04, *p* = 0.017) (Figure 3B).

### 3.5. ROI-Based Analysis of the Cervical Spinal Cord

**Figure 4:**
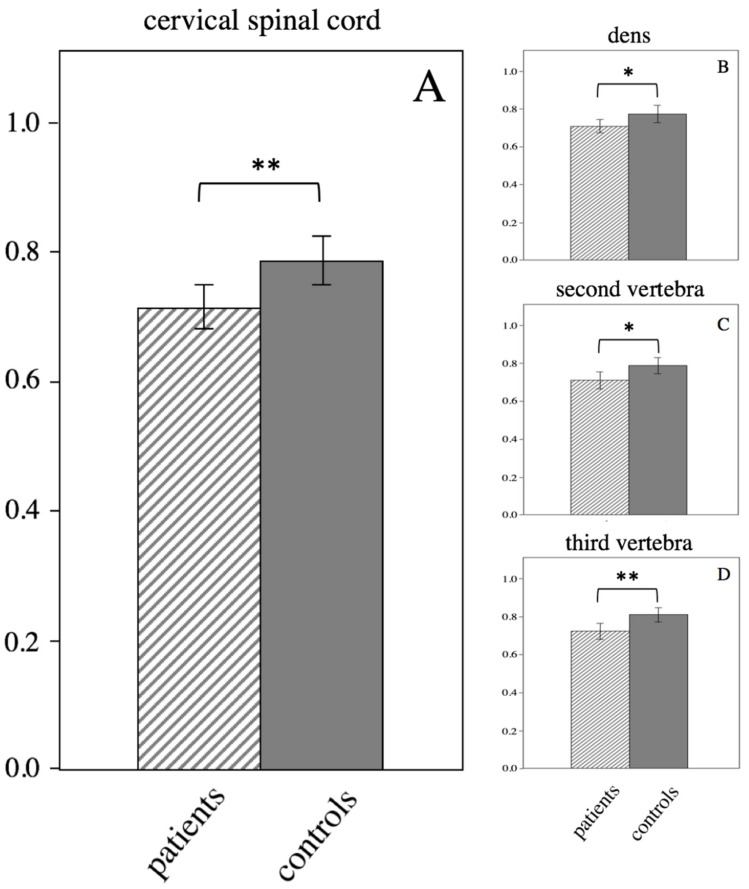
Decreased FA in the cervical spinal cord in HSP compared to controls. Error bars indicate 95% CI. Asterisk indicate *p* < 0.05, double asterisks *p* < 0.01. Significantly lower FA values in the cervical spinal cord (A) and at the level of the dens (B), the second vertebra (C) and the third vertebra (D).

The cervical spinal cord was analyzed at level of the dens and the center of the body of the second and third cervical vertebra. At all levels, a significantly reduced FA was found in pHSP patients compared to healthy controls (Figure 4). FA was reduced at the level of the dens (patients vs. controls 0.71 ± 0.07 vs. 0.77 ± 0.09, *p* = 0.045), the second vertebra (0.71 ± 0.10 vs. 0.79 ± 0.08, *p* = 0.022) and the third vertebra (0.72 ± 0.09 vs. 0.81 ± 0.08, *p* = 0.003). Average FA of the cervical cord was also reduced (0.72 ± 0.07 vs. 0.79 ± 0.07, *p* = 0.003).

### 3.6. Correlation Analysis between DTI Parameters and Clinical Data

**Figure 5:**
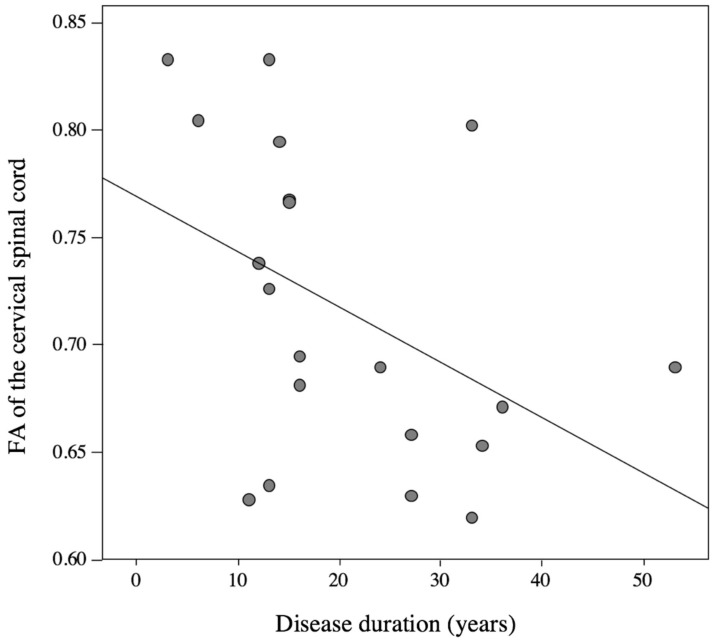
Correlation between FA of the cervical cord and disease duration. FA of the cervical cord correlates significantly with disease duration of pHSP patients (*r* = −0.457, *p* < 0.05).

Correlation analysis of FA values with clinical data revealed a significant negative correlation of the FA value of the cervical spinal cord with disease duration of pHSP patients (Spearman *r* = −0.457, *p* < 0.05) (Figure 5). No correlation was found between further clinical data, e.g., age and disease severity (assessed by the spastic paraplegia rating scale) and DTI parameters. Correlation analysis was performed using the FA values obtained by ROI measurements.

## 4. Discussion

Diffusion tensor imaging represents an advanced imaging technique that has been shown to be suitable for analyzing structural changes in rare motor neuron diseases [28]. We used this approach to visualize and identify structural white matter alterations in pHSP patients. While conventional MR imaging in these patients is useful for excluding other diseases, DTI can reveal structural damage of white matter tracts [29]. Tract-based voxel-wise DTI analysis of pHSP patients using TBSS revealed structural changes of the corticospinal tract in pHSP. As expected regarding the pathophysiology of pHSP, FA reductions were present at most brain levels of the CST (centrum semiovale, internal capsule and cerebral crura) overlapping with an increased AD and MD. Widespread FA decrease of the CST was described by several recent studies [14,18,30] including Lindig et al [20] who demonstrated alteration of white matter integrity of the CST of 15 SPG4 patients using TBSS. These findings are consistent with the hypothesis of a dysfunction of upper motor neurons and the CST as predominant cause of spasticity and weakness of the lower limbs [2].

Alterations of brain parenchyma diffusion indices were not limited to motor tracts. Moreover, our results suggest a more widespread and diffuse microstructural damage of white matter tracts in HSP. A broad impairment of white matter tracts including association, commissure and projection fibers could be demonstrated. In addition to the CST, FA reductions were also found in primary non-motor regions like the corpus callosum, the fornix, the corona radiata as well as in most association fibers including the superior and inferior longitudinal fasciculi, the inferior fronto-occipital fasciculi and the cingulum. These are expected findings in complicated HSPs as SPG11 showing additional symptoms and indicate a clinically perceptible impairment beyond the motor system and match the results of studies of SPG11 patients that revealed pathological changes and atrophy of grey and white matter—especially of the corpus callosum [15,16,31,32]. The broad alterations in pure HSP found in our study confirm findings of previous studies analyzing multiple genotypes of pure and complicated HSP (e.g., SPG3a, 4, 5, 7, 10, 11, 15, 31, 35) [14,33] and studies concentrating on SPG4 [18,20] showing a widespread impairment of commissure, association and projection fibers dominated by the corpus callosum and the CST as well. Aghakhanyan et al [30] demonstrated white matter involvement in 12 pHSP patients performing TBSS. They found widespread FA reduction in several brain regions including the CST, the corpus callosum, inferior and superior longitudinal fasciculi and anterior thalamic radiations.

The extent of the alteration of white matter fiber tracts beyond the motor system suggests that the disease may not only be explained by pathological changes of the CST. The appearance of non-motor symptoms such as depression, fatigue or pain in SPG4 patients described by Servelhere et al [34] indicates neuronal damage beyond the CST. Even SPG4 patients that tend to be classified as pure HSP sometimes show additional symptoms like ataxia, cognitive impairment and lower motor neuron dysfunction [35], pointing to pathological changes of non-motor systems. A previous study [36] addressing the cellular pathology of the disease, demonstrated that spastin, mutated in SPG4 HSP, is widely expressed in neurons of the central nervous system and is not limited to CST neurons and axons. Furthermore, tau pathology as an indicator for cytoskeletal dysfunction was not only found in spinal cord motor neurons but in non-motor regions as well [36]. These cellular anomalies of the neuroglia also suggest that the disease is not limited to the motor system [36].

The combination of the DTI indices fractional anisotropy, radial diffusivity, axial diffusivity and mean diffusivity can help reveal pathophysiological features of the disease as they characterize different patterns of microstructural change across brain tissues, in particular the differentiation between axonal damage and changes in myelination of neuronal fibers. DTI metrics were not normally distributed in the corpus callosum, the internal capsule, the cerebral crura and the cervical spinal cord suggesting a disease specific degeneration pattern. FA reduction is generally regarded as surrogate parameter of impaired microstructural integrity whereas AD increase indicates axonal damage and RD increase demyelination of neuronal fibers [29,37,38,39]. In our study, reduced FA was detected in white matter tracts beyond the CST. To a lesser extent, AD and MD were increased in the corpus callosum, parts of the semioval center, the CST and several association fibers. No difference of RD could be found between pHSP patients and healthy controls. The decrease of FA suggests a widespread alteration in microstructural integrity of white matter tracts of HSP patients. Moreover, FA decrease combined with AD and MD increase indicates axonal damage as predominant pathophysiological change. Axonal degeneration is the most common explanation for the characteristic symptoms of HSP patients [40], probably caused by several molecular degenerations, e.g., abnormal axonal transport, endosome membrane trafficking, endoplasmic reticulum morphology, protein misfolding or mitochondrial disturbance depending on the exact mutation [1,40]. These pathogenetic abnormalities may cause a degeneration or abnormal development especially of the long spinal cord axons leading to lower extremity spastic weakness as a main symptom of the disease [40].

The most severe alterations in FA were detected in the cervical spinal cord of pHSP patients. Our analysis revealed significantly reduced FA at the level of the dens, the second and third cervical vertebra. Only a few previous studies have analyzed the spinal cord of HSP patients so far and were focusing merely on the volume-loss of the spinal cord [12,14,18,21]. Common result of these studies was an atrophy of the spinal cord. However, specific analysis of the lateral funiculus as part of the CST has not been performed. We focused on the lateral funiculus containing the lateral corticospinal tract with a ROI-based approach to proof an involvement of the spinal CST. Postmortem studies of HSP patients demonstrated axonal degeneration of the CST, predominantly in the spinal cord with a maximum in the distal parts of the neuronal fibers [36,41,42]. The long axons of the CST seem to be affected most as they are more vulnerable, therefore supporting the hypothesis of a “dying back” axonopathy [1,41]. In our study, the cervical parts of the CST appeared to be more affected than the intracranial parts matching the concept of an ascending degeneration of the CST [41].

The limitations of our study comprise the incapacity of clinical spine coils to carry out DTI measurements beyond the level of the third body of the cervical spinal cord. Signal loss caudal of C3 and respiratory movement artifacts hamper correct identification of the relevant structures. Therefore, future studies performing DTI of the whole spinal cord are needed to confirm the concept of an ascending degeneration by demonstrating a primary impairment of caudal parts of the CST in HSP patients. Potentially, DTI of the spinal cord could provide useful information in early HSP about the pathophysiology of the disease.

Our results indicate a retrograde axonal degeneration of the CST mainly affecting the distal parts of the axons of pyramid neurons. Hereby the significant negative correlation of FA of the cervical spinal cord with disease duration indicates that a longer disease duration is related to a reduced FA value. This suggests that FA may serve as a suitable progression marker to monitor clinical and subclinical structural changes. Further longitudinal studies and long term follow-ups will be necessary to confirm this hypothesis.

## 5. Conclusions

DTI analysis of the corticospinal tract from the internal capsule to the cervical spine reveals reduced FA in pure hereditary spastic paraplegia and indicates microstructural damage and axonal degeneration of motor neurons. The CST at the level of the cervical spinal cord is thereby more severely affected than the intracranial part of the CST, suggesting an ascending axonal degeneration of the CST. The significant negative correlation of FA of the CST at the level of the cervical cord with disease duration indicates that FA may serve as a future progression marker for assessment of the disease course in HSP

## Figures and Tables

**Table 1:** Demographic and clinical characteristics of hereditary spastic paraplegia (HSP) patients and control subjects.

Clinical Information		Patients with pHSP (*n* = 20)	Controls (*n* = 17)	*p*-Value
**Age**		52.7 ± 9.7	55.0 ± 8.9	0.45
**Gender (female)**		60.0%	64.7%	0.82
**Disease onset (year)**		32.0 ± 15.2		
**Disease duration (year)**		20.7 ± 12.3		
**Genetic mutation:**	SPG4 SPG5 SPG31 unknown	10 (50%) 2 (10%) 1 (5%) 7 (35%)		
**SPRS score**	total	17.7 ± 7.2		
	subitem A	11.0 ± 4.4		
	subitem B	3.9 ± 1.8		
	subitem C	2.8 ± 2.0		

pHSP = pure form of HSP, mean ± SD: data are means ± standard deviations, SPRS = spastic paraplegia rating scale (52 points), Subitem A: Functional score (24 points), Subitem B: Spasticity and weakness (16 points), Subitem C: Additional symptoms (12 points). *p*-values show group comparison using two-sample *t*-test or Mann-Whitney U test depending on the normal contribution.

**Table 2:** Fractional anisotropy (FA) in HSP patients and controls.

Anatomical Region	Patients	Controls	*p*-Value
corpus callosum	0.84 ± 0.05	0.87 ± 0.05	**0.048**
corpus callosum splenium	0.86 ± 0.04	0.90 ± 0.04	**0.017**
corpus callosum rostrum	0.81 ± 0.06	0.85 ± 0.06	0.104
internal capsule	0.77 ± 0.06	0.80 ± 0.06	**0.048**
cerebral crura	0.80 ± 0.06	0.82 ± 0.06	0.209
cervical spinal cord	0.72 ± 0.07	0.79 ± 0.07	**0.003**
cervical spinal cord dens	0.71 ± 0.07	0.77 ± 0.09	**0.045**
cervical spinal cord second vertebra (body)	0.71 ± 0.10	0.79 ± 0.08	**0.022**
cervical spinal cord third vertebra (body)	0.72 ± 0.09	0.81 ± 0.08	**0.003**

Statistical analysis of the fractional anisotropy (FA) in hereditary spastic paraplegia (HSP) patients shows significant decrease in the corpus callosum and cervical spinal cord in comparison to healthy controls. Data are means ± standard deviations, *p*-value calculated using Mann-Whitney U test, significant *p*-values (<0.05) in bold print.

## Abbreviations

1.	Hereditary spastic paraplegia (HSP)
2.	High angular resolution diffusion tensor imaging (HARDI-DTI)
3.	Tract-based spatial statistic (TBSS)
4.	Fractional anisotropy (FA)
5.	Corticospinal tract (CST)
6.	Spastic paraplegia (SPG)
7.	pure HSP (pHSP)
8.	Region-of-interest (RO)
9.	Spastic paraplegia rating scale (SPRS)
10.	echo planar imaging (EPI)
11.	NIfTI files (Neuroimaging Informatics Technology Initiative)
12.	FMRIB Diffusion Toolbox (FDT)
13.	standard deviation (SD)
14.	interquartile range (IQR)
15.	Axial diffusivity (AD)
16.	Mean diffusivity (MD)
17.	Radial diffusivity (RD)
